# Address

**Published:** 1873-07

**Authors:** 


### ADDRESS.

*Of Dr. W. H. Goddard, upon retiring from the Presidency of the Kentucky State Dental Society, at Louisville, June 5th,* 1873.

"It has become a time-honored custom, which I would not disregard, for the presiding officer, on retiring from the chair, to address his associates on some topic pertaining to our profession, so that his latest official act shall be an effort to arouse in their minds a fresh measure of interest in their profession of itself, and in this association, as a means of culture for its requirements, and of elevating and stimulating social fraternity. Before I announce the subject which I have selected, in order to carry out this pleasant but responsible duty. I take occasion to congratulate you, gentlemen, on the happy auspices under which we have assembled together at this our annual meeting. During the interval since last we met not one of our number has been summoned away from the duties and enjoyments of earth to the higher and better life beyond the grave; while the great majority, I believe, have reason to thank the kind Giver of all good for innumerable blessings which he has showered upon their pathways. Let us hope that those of us who have been subjected by that same loving Providence to seasons of trial and discipline, if there be any such, have had grace given them to descry sources of blessing in even such occasions of suffering, so that we can all join in offerings of devout thanksgiving, and in vows of self-consecration to truth and duty, and thus deserve the utmost benedictions of paternal love in the unknown future. Gentlemen, I shall not detain you long; and I have not vexed my brains in an effort to strike out an original and novel train of remark. On the contrary, I shall employ some of the most familiar associations of our profession in the past, as a stimulus to a higher ambition and an ever increasing ardor of interest and effort in the future.

I shall speak of the Spirit of Progress of the present age as it has given impulse and character to our profession.

It is very hard to pronounce upon the attainments and progress of any age of the world in comparison with another, because the conditions of thought and action in every two such instances are so peculiar and contrasted, that what may seem in some lights to be feeble and puerile, in others deserve to be rated as gigantic and marvelous. And yet, I think tha this nineteenth century, with its railroads and steamships, its scientific researches, and that miracle of intercourse by which the "Puck" of Shakespeare, who said he would put a girdle round the earth in forty minutes—is beaten out and out, and a girdle put around the earth with the speed of thought itself, deserves to be rated as being especially the age of progress.

Some writers tell us that the ancients knew more of some things than we do; and talk about "the lost arts." Very likely there were artistic processes, some of them very curious and beautiful, which we do not know how precisely to emulate. But it is when we bring the triumphs of the Nineteenth century in art and science home to the hearts and lives of the masses of the people, facilitating intercourse, bringing the nations into immediate contact as one family, dispelling ignorance, putting machinery in the place of human muscle and sinews, renovating commerce, reconstructing society and uplifting the individual, it is then that the immense superiority of the present age becomes manifest. "The lost arts" of the ancients pertained to the luxuries of the high born alone. The arts of to-day enable nation to greet nation in friendly embrace, and illustrate the finality of the great federal compact of universal nature.

These results are not confined to any special pathways of human endeavor, or any particular branches of science, but with wonderful universality, affect all departments of science and art, and all the avocations of life alike. The first principles of everything, both in general and in detail, are sought after with unwearied diligence and consummate sagacity, and every walk and profession, enabled thereby to understand its own capacities and opportunities, is rapidly marching on along the open avenues to perfection.

Restricting the subject to our own profession, I remark that dentistry being intrinsically a branch of surgery, I am led to call your attention to the astonishing fact in relation to surgery in general, that now the terrors once pertaining to this thrice-honorable department of practical science are all done away by the means we have of utterly negativing the pain it inflicts.

The world is not half alive to—not half grateful enough for this singular blessing. Once the amputation of a limb, or the removal of a tumor was looked forward to by the patient as something to be dreaded worse than death itself; and was resorted to, for the most part, only when the alternative was the operation or speedy decease. Science has removed all these pangs, and thrown the angelic mantel of oblivion around the patient, who sweetly sleeps in unconsciousness while the instruments of the surgeon are carving through his flesh. So too, death itself, as sometimes it makes its slow and torturing approaches, is robbed of its fearful sting. Even the gallows has enjoyed the immunity imparted by science, and the criminal been rendered unconscious of the horrors of his taking off. Whether this is not pushing open the door of advantage a little too far, I will not delay to discuss.

No other branch of practical science has surpassed that of dental surgery in the marvel of its progress. During the seventeenth century it made but few advances, and was in a very crude and elementary condition. But in the eighteenth century it took a noble start. It became a subject of more critical inquiry than before, and of thorough investigation according to the measure of the culture and appliances of the time. Eminent men wrote on the subject, and a broad and deep foundation was laid, on which other eminent men have subsequently reared a noble and commanding superstructure. Dentistry at length began to attract general attention, and to assume a distinct character as a profession. Still its progress was slow. At the commencement of the present century a brighter era in this department of surgery began, and from thenceforth its progress was destined to be rapid. For it began to feel the stimulus of the spirit of enterprise and improvement which was stirring men to the very centers of power, and nerving them to new and grander achievements in every department of science and art. Many noble minds, animated by a spirit of emulation, devoted their time and talents to the furtherance of this particular branch of science. They went bravely and triumphantly on, undeterred by whatever obstacles, toward the summit of professional excellence.

From that time, many elaborate treatises on the subjec. have been issued from the press in different countries, which have been translated severally into other languages, and diffused in every civilized land.

In our own country, dental surgery as a distinct and progressive profession is of recent origin. It was first introduced as such during the time of the revolutionary war, but when it had once obtained a foothold, it enlisted in its interests and advancement some of the finest abilities and choicest practical skill, of which our people can boast so much ; and before long we find American dentists surpassing those of Europe in the mastery of that combination of principles and practice which forms the basis of the proudest researches in science and art, in some instances supplanting their European contemporaries in the fields of their own effort and enterprise.

But it is within the last quarter of a century, or a little more, that dentistry has advanced to its present perfection. And as it grew in repute and favor, as it distributed garlands of honor among its prominent practitioners, and promised ample emolument for eminent success, it shared the fate of all successful human enterprises and undertakings, in gathering to itself a host of ignorant pretenders and charlatans, eager to reap large pecuniary benefits under its protecting ægis. And while, on the other hand, we had a Hudson, a Haydon, a Koecker, a Harris, and many other distinguished men to give the profession standing and impetus through their abilities and labors in its behalf, on the other hand we had hundreds who rushed with brazen effrontery through the open portals and assumed the insignia of the profession, while destitute of any qualifications for its successful and honorable prosecution.

The dangers which menaced it in this direction led to a new phase in its condition, which was destined to give it fresh character and position. Some of its bright ornaments, who felt it to be necessary to enable the community to discriminate between the competent and the incompetent of those who had entered the profession, and thus to prevent gross imposition, who also appreciated the additional advantage of elevating the standard of professional capacity and sufficiency, and affording the means of progress in professional culture, formed themselves into State associations and district societies for the interchange of thought and mutual improvement. Only those fitted by character and culture to discharge the high and responsible duties incident to the profession have been admitted to this noble brotherhood.

This was a step in the right direction. It sifted the chaff from the wheat. It inspired the members of the associations thus formed, with fresh enthusiasm for their calling and an eager spirit of inquiry for further and grander advances. It spurred the science of dental surgery rapidly forward ; developing minds hitherto quiescent into admirable activity, to reap large results in every department of the profession.

The spirit of progress did not end its energizing influences here. Still newer and untried sources of improvement were suggested and demanded by the bright lights of the profession, and dental colleges were instituted to which students could resort and obtain a thorough medico-dental education ; and thus carry with them at the beginning of their career a passport to the confidence of the public.

Within the past few years how rapid has been the advance of our profession, and is not this greatly due to the influence of the associations which exist among us ?

When the members of such associations, annually or oftener, assemble together for interchange of ideas, and freely impart one to another, the results of their thought, investigations and experience, must not a new impetus be acquired from this accumulation of the sources of power and progress ? Believe me I do not exaggerate the importance of such affiliations. Our own association, although young, and, I am sorry to say, few as yet in numbers, may become the source of incalculable beniefit, f its opportunities are appreciated and wisely improved

If laying aside all jealousies and personal rivalries, and the bickerings and estrangements which they generate, all seek to obtain the good which may be conferred, in the interests of individual improvement, of the advancement of science, and of the honor and progress of the profession.

I will not delay to recount the important improvements which have been made of late in material, instruments, and mechanical appliances to facilitate our operations.

I will only suggest that this constitutes a highly interesting field of observation of itself which I trust some one will survey for our advantage.

Such is a rapid review of the results of the spirit of progress as it has developed the scope and resources of our profession. And recurring once more to the immense advantages which have been derived from association, I beg you, gentlemen, to bring these remarks to bear, as they have been earnestly intended, on the interests of our own association, and the duties which it imposes. Are any indifferent to the sources of power and progress which it places in our possession? Are any careless of the promotion and diffusion of dental science, which similar associations have accomplished? Are any unmoved by the recorded history of triumphs effected through such agencies? Are any willing to hold themselves aloof from co-operative effort, and say that they have no individual concern with the subject?

Gentlemen, much has been gained, but let none be satisfied. Far greater advances and successes are yet in store. While all other sciences and arts are constantly striding forward with giant steps, our own profession has an equally open field for fresh attainments; and it is for us to lend it all the stimulus and energy to be realized from our own devoted and zealous co-operation, as it careers forward towards the exalted summit of perfection. There is everything to animate us. We have seen what results have ensued from the endeavors of single individuals; and the power of association is after all only the effect of individual resources brought into one aggregate, and gaining strength from interdependence and centralized energy. Then let us bend every effort to fulfill our part in the noble work, and become helpers towards the great consummation.

Let us not be content with doing nothing to retard it. Let us not stand indifferent, mere curious lookers-on! But when the close of the nineteenth century shall exhibit, as it surely will, a record of knowledge acquired, of discoveries made, of inventions perfected, of skill exhibited, such as would astonish us, were it displayed to-day, so far will it surpass not only the actual searches of the profession, but the very dreams of its enthusiastic disciples; let it be that the issues of our interest and our diligence shall have entered as materials and forces, in the production of the grand result: and though no special applause may accrue to us among our fellow-men, though our work may be too humble to win for us renown, we shall go down to our graves in the happy consciousness of duty performed, of energies exerted, of our chosen profession cherished and honored in our characters, our culture, our interest, our skill, and the world be made better for that we have lived!"

